# A primary care-oriented framework for osteoporosis prediction: integrating multi-algorithm feature selection and machine learning model benchmarking

**DOI:** 10.3389/fendo.2026.1917386

**Published:** 2026-10-05

**Authors:** Xiaofang Guan, Xiaoqi Wang, Wenjing Zhang, Juanjuan Li, Zhili Hou, Zhaoting Hu, Pei Sun

**Affiliations:** Health Management Center, the Third Affiliated Hospital of Southern Medical University, Guangzhou, China

**Keywords:** dual-energy X-ray absorptiometry, machine learning, osteoporosis, predictive model, primary care

## Abstract

**Summary:**

Large-scale screening and primary care diagnosis of osteoporosis are challenging due to the limited availability of dual-energy X-ray absorptiometry (DXA). Therefore, predictive models for osteoporosis that do not rely on bone mineral density measurements are essential. Using routine health examination data and applying a consensus feature selection strategy, we constructed machine learning (ML)-based models and compared their performance across multiple dimensions.

**Purpose:**

To develop a robust, low-cost, and interpretable framework for osteoporosis prediction based on readily available information, which can be easily integrated into electronic health systems to inform DXA referral in resource-limited settings.

**Methods:**

Variables covered by the basic health examination package were obtained from participants who underwent health examinations between January 2024 and January 2025. We implemented a strategy in which only features selected by at least four out of the seven feature selection methods were retained. Six ML algorithms were employed to develop models, and their prediction performances were compared across discrimination, goodness-of-fit, and clinical utility.

**Results:**

A total of 2,260 participants were included, with an osteoporosis prevalence of 7.1%. Eight features: age, sex, body mass index, alkaline phosphatase, hematocrit, high-density lipoprotein, urea, and platelet count, were selected for model development. In the validation set, the random forest and artificial neural network (ANN) models demonstrated strongest screening performance, with AUCs of 0.895 (95% CI 0.867–0.923) and 0.890 (95% CI 0.862–0.919), respectively. The ANN model additionally exhibited superior calibration. Shapley additive explanations were applied to interpret the ANN model for osteoporosis prediction.

**Conclusion:**

This study presents an easily integrable model for osteoporosis prediction based on routine health examination data. The ANN model provides robust prediction, offering a potential tool for pre-DXA triage and early identification of high-risk individuals in primary care and resource-limited settings.

## Graphical Abstract

**Figure:**
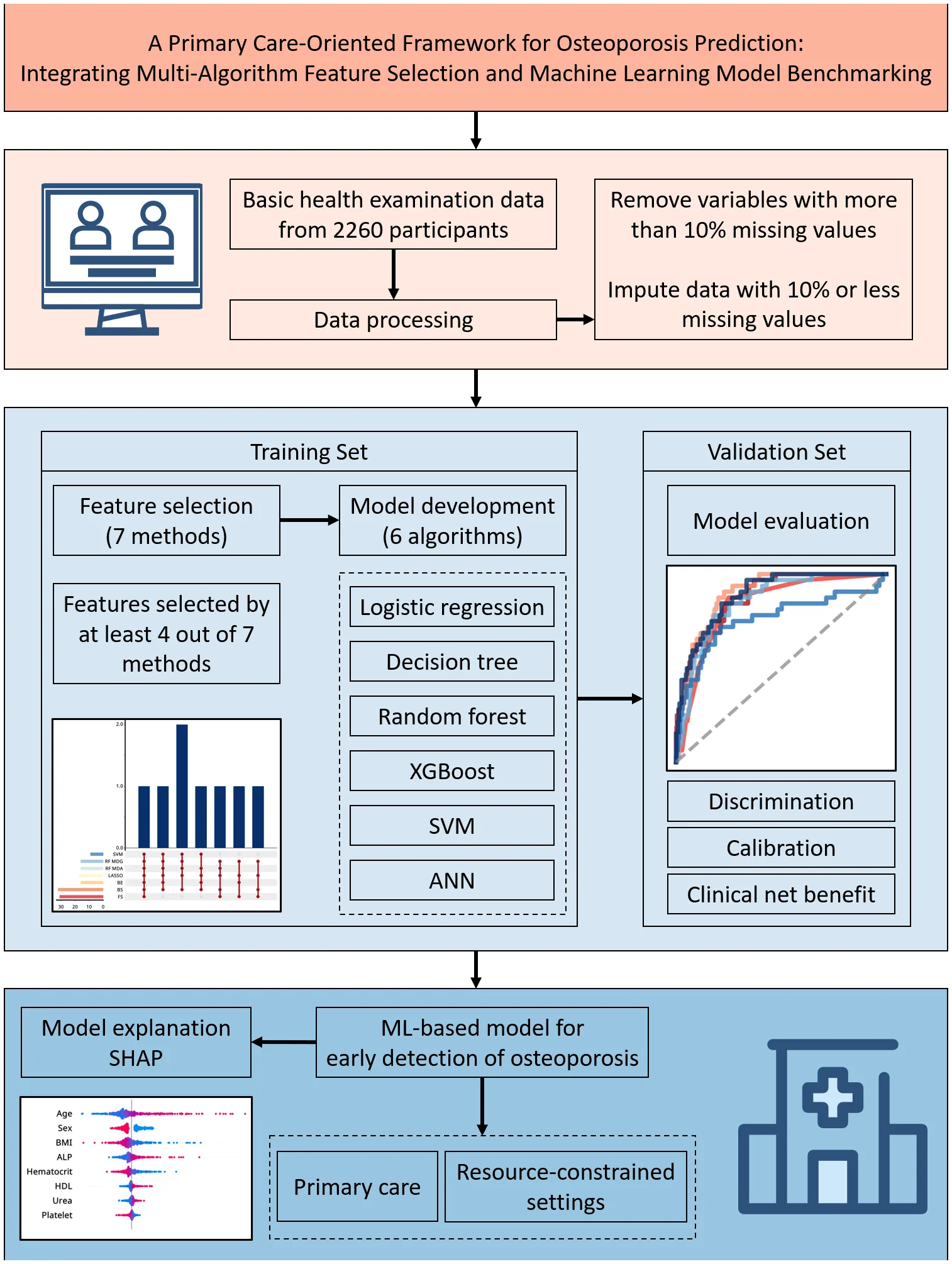

## 1 Introduction

Osteoporosis is a systemic skeletal disease characterized by decreasing bone mass and microarchitectural deterioration of bone tissue, leading to increased bone fragility and a consequent increase in fracture risk (1). The global prevalence of osteoporosis has been estimated at 18.3% to 19.7% (2, 3). In China, the prevalence among those aged 40 years or older was 20.6% in women and 5.0% in men (4). This major global public health challenge imposes an escalating burden amid the progression of population aging. Therefore, early identification of individuals at high risk for osteoporosis is a health care priority, enabling targeted interventions such as bone mineral density (BMD) assessment and timely anti-osteoporotic treatment to prevent fractures.

The current diagnostic gold standard for osteoporosis relies on dual-energy X-ray absorptiometry (DXA) to measure BMD (5, 6). However, the cost and limited accessibility of DXA services restrict its widespread implementation, especially in resource-constrained settings and primary care hospitals. Additionally, large-scale screening of BMD is impractical due to concerns about radiation exposure (7). Consequently, effective pre-screening tools are essential to identify high-risk individuals who would benefit most from confirmatory DXA, thus optimizing resource allocation. Although several preclinical screening tools have been developed to predict osteoporosis (8, 9), their clinical utility remains limited by suboptimal accuracy and vulnerability to patient recall bias and reporting variability.

Artificial intelligence has achieved remarkable results throughout medicine (10–12). Machine learning (ML) algorithms excel at identifying complex patterns within multidimensional and nonlinear data and have demonstrated significant potential in disease prediction and risk stratification (12). Recent studies have begun exploring ML models for osteoporosis prediction (13–16), yet critical considerations remain regarding the clinical accessibility of input parameters, the availability of external validation, the frequent underreporting of model calibration and clinical utility metrics, and the robustness of feature selection strategies.

In China, routine health examinations for employees and older adults regularly capture demographic and basic laboratory measures. Prediction tools leveraging these readily available features hold significant potential for large-scale osteoporosis screening. Even when individual parameters fall within normal reference ranges, ML algorithms enable autonomous detection of latent patterns within such complex datasets.

Here we developed a primary care-oriented framework for osteoporosis prediction utilizing routine health examination data. By integrating multiple ML algorithms for feature selection and predictive model construction, this framework enables early identification of high-risk individuals. It has the potential to support accessible triage for subsequent DXA screening decisions, particularly in hospitals with limited DXA availability, although its clinical utility and generalizability across different healthcare settings require further external validation.

## 2 Methods

### 2.1 Study population

This study consecutively enrolled individuals who underwent health examinations at the Third Affiliated Hospital of Southern Medical University between January 2024 and January 2025. The inclusion criteria were (1) being aged 18 years or older; (2) completion of a DXA scan; (3) ability to understand and being willing to participate in the study. Exclusion criteria were (1) recent examinations involving barium, iodine, or other contrast agents; (2) pregnancy; (3) history or current diagnosis of conditions affecting bone density (including but not limited to metabolic bone diseases or malignancies). Sample size determination was performed using the ‘pmsampsize’ package in R based on the number of candidate predictors. Before feature selection and model construction, the dataset was stratified according to osteoporosis status when divided into training and validation sets. Aiming to preserve the real-world data distribution and avoid generating synthetic patients, Class imbalance was addressed by threshold optimization rather than resampling or class weighting, in order to preserve the calibration of the predicted probabilities.

This study was conducted in accordance with the Declaration of Helsinki and approved by the Ethics Review Board of the Third Affiliated Hospital of Southern Medical University (2022-020).

### 2.2 Data collection

To ensure all predictors included are easily ascertained and readily available in different clinical scenarios, only parameters covered by the basic health examination package were included. Demographics, physical measurements, and hematologic, biochemical, and urinalysis parameters of the participants were retrospectively collected.

BMD was measured with a DXA scanner (Lunar iDXA, GE Healthcare). Measurements were performed at the axial skeleton, specifically the lumbar spine (L1-L4) and total hip or femoral neck. According to the World Health Organization criteria (17), participants were classified based on the lowest T-score at any measured site as: non-osteoporosis (T-score > -2.5) or osteoporosis (T-score ≤ -2.5).

### 2.3 Feature selection

Prior to model development, ML-based feature selection algorithms were applied to the training set to identify optimal predictors. To enhance robustness of the framework, we implemented a strategy in which only features selected by at least four out of seven algorithms were retained (18). These methods were chosen based on their proven effectiveness in handling high-dimensional data and identifying the most relevant predictors in predictive modeling.

Stepwise regression (SR) iteratively adds or removes predictors based on a predefined criterion (e.g., statistical significance), and can be used to obtain a parsimonious set of variables. However, SR does not inherently resolve multicollinearity; when predictors are highly correlated, variable inclusion may be unstable and should be interpreted with caution. We implemented three variants: forward selection (FS), backward elimination (BS), and bidirectional elimination (BE).

The least absolute shrinkage and selection operator (LASSO) applies an L1 penalty to reduce the coefficients of less important predictors toward zero, thereby selecting a sparse subset of relevant features and reducing the risk of overfitting.

For the random forest (RF) method, we employed two importance measures: mean decrease accuracy (MDA) and mean decrease in Gini impurity (MDG). RF was selected for its robustness in modeling nonlinear relationships and interactions among predictors, as well as its capacity to rank feature importance in relation to the outcome.

Support vector machine (SVM) was employed for its iterative strategy of removing the least important features to optimize subset selection based on model performance, thereby ensuring that the retained features are highly relevant to the classifier.

### 2.4 Model development and hyperparameter tuning

ML model development was performed exclusively within the training set. Six ML algorithms were applied: logistic regression (LR), decision tree (DT), RF, extreme gradient boosting (XGBoost), SVM, and artificial neural network (ANN).

Hyperparameters were tuned by randomized search within the training set. For each of the six algorithms, 100 configurations were drawn independently from pre-specified distributions and evaluated by stratified 5-fold cross-validation. Model selection was guided by the mean cross-validated ROC-AUC as the primary metric, chosen because it is threshold-independent and invariant to class prevalence, which permits direct comparison across algorithms and resampling schemes. PR-AUC, the Brier score, and sensitivity and specificity at the Youden-optimal threshold were examined as secondary metrics. PR-AUC was included because it is sensitive to the relatively low prevalence in our cohort. Feature standardization and the consensus feature-selection procedure were performed within the training set before cross-validation; the cross-validated AUC therefore served as an internal optimization criterion. The validation set was excluded from all preprocessing, feature-selection, and tuning steps, so the final validation set estimates remain unbiased. Seeds were fixed for data splitting, hyperparameter sampling, and model initialization. For the ANN, the retained configuration comprised a single hidden layer of 32 units with tanh activation, trained with Adam (alpha = 0.0890, learning_rate_init = 0.00608, batch_size = 64, beta_1 = 0.85, beta_2 = 0.999), with early stopping enabled.

### 2.5 Model evaluation and bootstrap analysis

Model performance was assessed using the validation set across three dimensions: discrimination, calibration, and clinical utility.

Discriminative ability was evaluated using a comprehensive set of metrics, including sensitivity, specificity, area under the receiver operating characteristic curve (AUC), optimal cutoff value, positive and negative likelihood ratios (PLR and NLR), positive and negative predictive values (PPV and NPV), and the F1 score (precision and recall). Statistical comparisons of AUCs among models were conducted using the DeLong test. The Osteoporosis Self-Assessment Tool for Asians (OSTA) score was also calculated for benchmark comparison.

Calibration, the agreement between predicted probabilities and observed outcomes, was assessed via calibration plots and Brier scores, providing insight into each model’s reliability in probabilistic risk estimation. Clinical utility and net benefit were quantified through decision curve analysis (DCA).

To enhance the interpretability of complex and non-transparent (“black-box”) models, SHapley Additive exPlanations (SHAP) analysis was employed. SHAP is a game-theoretic method that quantifies the contribution of each input feature to individual predictions by computing Shapley values, thereby improving transparency for potential clinical translation.

To assess robustness beyond a single random split, we conducted bootstrap resampling with out-of-bag evaluation. For each of 400 iterations, we drew a bootstrap sample as the training set. Feature selection, model fitting, and threshold selection were performed exclusively within the bootstrap training sample. Performance was then evaluated on the out-of-bag observations. Sensitivity analysis of the consensus criterion was assessed by repeating the procedure under three predictor sets, comprising the variables retained by at least three, at least four, or at least five of the seven selection methods (24, 8 and 6 variables, respectively).

### 2.6 Statistical analysis

Variables with more than 10% missing values were removed for data processing, whereas those with 10% or less were imputed using Multiple Imputation by Chained Equations (MICE).

Continuous variables with normal distribution were expressed as mean (standard deviation [SD]) and compared with the Student’s *t* test. Those with non-normal distribution were expressed as median (interquartile range [IQR]) and compared with the Mann-Whitney U test. Categorical variables were expressed as counts and percentages, and compared with the chi-square test or Fisher’s exact test, as appropriate. Two-sided *P* values <0.05 were considered statistically significant.

Statistical analyses were performed using R software (version 4.4.2) and Python (version 3.10.13).

## 3 Results

### 3.1 Baseline characteristics

A total of 2,260 participants were included in the analysis, with osteoporosis diagnosed in 160 individuals (prevalence 7.1%). The median age in the osteoporosis cohort was 64 years (IQR 58-70), and 136 (85.0%) were female. Demographic characteristics and laboratory parameters from health examinations are detailed in **Table 1**. Participants were randomly allocated to training (80%) and validation (20%) sets, with balanced outcome distributions across both sets. Baseline characteristics of both sets are presented in **Supplementary Table S1**. The validation set was rigorously excluded from all feature selection and model training processes.

**Table 1:** Baseline characteristics.

Variables	Non-osteoporosis (n=2100)	Osteoporosis (n=160)	P value
Age (years)	54.00 (48.00, 61.00)	64.00 (58.00, 70.25)	**<0.001**
Sex, n (%)			**<0.001**
Female	911 (43.4)	136 (85.0)	
Male	1189 (56.6)	24 (15.0)	
BMI (kg/m^2)	24.03 (22.08, 26.21)	22.13 (19.76, 23.73)	**<0.001**
Hypertension, n (%)	480 (22.9)	70 (43.8)	**<0.001**
RBC (x10^12/L)	4.78 (4.46, 5.11)	4.50 (4.23, 4.87)	**<0.001**
HGB (g/L)	142.00 (132.00, 152.00)	133.00 (125.75, 141.00)	**<0.001**
MCV (fL)	90.50 (87.70, 93.00)	91.00 (88.57, 94.65)	**0.036**
MCH (pg)	30.10 (29.00, 31.00)	29.90 (28.99, 31.00)	0.274
MCHC (g/L)	331.00 (324.00, 338.00)	327.00 (321.00, 333.00)	**<0.001**
Hematocrit (%)	0.43 (0.40, 0.45)	0.41 (0.38, 0.43)	**<0.001**
Platelet (×10^9/L)	243.00 (208.00, 280.00)	235.00 (202.75, 270.25)	**0.040**
Plateletcrit (%)	0.24 (0.21, 0.27)	0.23 (0.20, 0.27)	0.079
MPV (fL)	9.80 (9.30, 10.40)	9.90 (9.47, 10.32)	0.297
WBC (×10^9/L)	5.92 (5.04, 6.95)	5.66 (4.80, 6.68)	**0.009**
Lymphocyte (×10^9/L)	1.99 (1.63, 2.39)	1.93 (1.53, 2.23)	**0.017**
Neutrophil (×10^9/L)	3.22 (2.62, 4.01)	3.13 (2.42, 3.82)	0.079
Eosinophil (×10^9/L)	0.14 (0.09, 0.22)	0.11 (0.08, 0.17)	**0.001**
Basophil (×10^9/L)	0.03 (0.02, 0.04)	0.02 (0.01, 0.03)	**0.002**
Monocyte (×10^9/L)	0.41 (0.33, 0.51)	0.39 (0.32, 0.47)	**0.012**
Na (mmol/L)	139.40 (138.60, 140.00)	140.00 (139.00, 141.00)	**<0.001**
K (mmol/L)	4.10 (3.95, 4.24)	4.09 (3.90, 4.23)	0.240
Ca (mmol/L)	2.36 (2.31, 2.41)	2.36 (2.31, 2.41)	0.906
Cl (mmol/L)	104.06 (103.00, 105.18)	104.30 (103.38, 105.30)	**0.025**
Uric Acid (μmol/L)	371.00 (313.00, 437.00)	333.00 (289.75, 385.25)	**<0.001**
Creatinine (μmol/L)	73.00 (62.00, 85.00)	63.70 (57.00, 72.00)	**<0.001**
Urea (mmol/L)	5.08 (4.30, 5.91)	5.44 (4.62, 6.11)	**0.001**
Glucose (mmol/L)	5.13 (4.73, 5.66)	5.26 (4.89, 5.79)	0.057
Total Cholesterol (mmol/L)	5.32 (4.66, 6.00)	5.46 (4.74, 6.29)	0.082
Triglycerides (mmol/L)	1.32 (0.97, 1.88)	1.19 (0.90, 1.57)	**0.004**
HDL (mmol/L)	1.34 (1.14, 1.55)	1.53 (1.28, 1.84)	**<0.001**
LDL (mmol/L)	3.41 (2.89, 3.88)	3.36 (2.94, 3.97)	0.926
Total Bilirubin (μmol/L)	13.20 (10.90, 16.20)	12.61 (10.67, 15.22)	0.106
Direct Bilirubin (μmol/L)	2.10 (1.70, 2.60)	1.94 (1.60, 2.34)	**0.029**
Indirect Bilirubin (μmol/L)	11.10 (9.10, 13.70)	10.60 (9.00, 13.00)	0.106
GGT (U/L)	20.00 (14.00, 31.00)	18.00 (13.00, 25.25)	**0.016**
AST (U/L)	21.00 (18.00, 25.00)	23.00 (19.75, 27.00)	**<0.001**
ALT (U/L)	19.30 (15.00, 27.00)	18.00 (14.00, 23.00)	**0.006**
ALP (U/L)	71.00 (60.00, 83.45)	82.10 (70.15, 97.25)	**<0.001**
Total Protein (g/L), mean (SD)	72.77 (3.67)	73.28 (3.54)	0.088
Albumin (g/L)	44.50 (42.50, 46.51)	43.80 (41.80, 45.79)	**0.006**
Globulin (g/L)	28.20 (25.80, 30.70)	29.25 (27.10, 31.70)	**<0.001**
Urine PH	5.50 (5.20, 6.50)	6.00 (5.50, 6.50)	0.064
Urine Protein (positive), n (%)	33 (1.6)	2 (1.2)	1.000
Urine Bilirubin (positive), n (%)	12 (0.6)	1 (0.6)	0.616
Urobilinogen (positive), n (%)	74 (3.5)	0 (0.0)	**0.009**
Urine Glucose (positive), n (%)	103 (4.9)	8 (5.0)	0.851
Urine Nitrite (positive), n (%)	26 (1.2)	13 (8.1)	**<0.001**
Urine Occult Blood (positive), n (%)	537 (25.6)	66 (41.2)	**<0.001**
Urine Ketone Bodies (positive), n (%)	36 (1.7)	2 (1.2)	1.000
Urine WBC (positive), n (%)	466 (22.2)	71 (44.4)	**<0.001**

Data are expressed as median (IQR) unless otherwise indicated.

The bold entries indicate that the P value is less than 0.05.

BMI, body mass index; RBC, red blood cell; HGB, hemoglobin; MCV, mean corpuscular volume; MCH, mean corpuscular hemoglobin; MCHC, mean corpuscular hemoglobin concentration; MPV, mean platelet volume; WBC, white blood cell; HDL, high-density lipoprotein; VLDL, very-low-density lipoprotein; LDL, low-density lipoprotein; GGT, gamma-glutamyl transferase; AST, aspartate aminotransferase; ALT, alanine aminotransferase; ALP, alkaline phosphatase.

### 3.2 Selected consensus predictors

The variables identified by each feature selection method are listed in **Table 2**. The seven methods selected 31, 32, 16, 17, 16, 16, and 9 predictors, respectively. **Figure 1** illustrates the overlap among predictors selected by the seven methods. Features selected by at least four methods were retained as final predictors: age, sex, body mass index (BMI), alkaline phosphatase (ALP), hematocrit, high-density lipoprotein (HDL), urea, and platelet count. A total of eight predictors were ultimately included for model development.

**Table 2:** Features selected by different feature selection methods.

Methods	Selected features
SR-FS	Age, Hypertension, RDWCV, MCHC, Platelet, Plateletcrit, MPV, LPR, PDW, WBC, Neutrophil, Monocyte, Na, K, Ca, Cl, Urea, Glucose, Total Cholesterol, Triglycerides, HDL, VLDL, LDL, GGT, AST, Albumin, Albumin/Globulin, Urine Bilirubin, Urobilinogen, Urine Glucose, Urine Ketone Bodies
SR-BS	Sex, Age, Hypertension, BMI, MCH, MCHC, Hematocrit, Plateletcrit, PDW, Eosinophil, Basophil, Monocyte, Monocyte Percentage, Cl, Creatinine, Urea, Triglycerides, HDL, LDL, Direct Bilirubin, Indirect Bilirubin, AST, ALT, ALP, Globulin, Albumin/Globulin, Urine Protein, Urobilinogen, Urine Nitrite, USG, Urine Occult Blood, Urine WBC
SR-BE	Sex, Age, BMI, RDWCV, MCV, MCH, MCHC, Platelet, LPR, Lymphocyte Percentage, Eosinophil, Ca, LDL, GGT, Globulin, Albumin/Globulin
LASSO	Sex, Age, Hypertension, BMI, Hematocrit, Platelet, Basophil, Na, Creatinine, Urea, HDL, Direct Bilirubin, AST/ALT, ALP, Total Protein, Urine PH, USG
RF-MDA	Sex, Age, BMI, HGB, RDW, Hematocrit, PDW, Lymphocyte Percentage, Ca, Triglycerides, HDL, AST, AST/ALT, ALP, Total Protein, USG
RF-MDG	Age, BMI, ALP, HDL, RBC, AST/ALT, Creatinine, HGB, Urea, Hematocrit, Total Protein, Globulin, Glucose, MCV, Lymphocyte Percentage, Platelet
SVM	Sex, Age, BMI, RBC, HGB, Hematocrit, ALP, Urobilinogen, Urine Nitrite

SR, stepwise regression; FS, forward selection; BS, backward selection; BE, bidirectional elimination; LASSO, least absolute shrinkage and selection operator; RF, random forest; MDA, mean decrease accuracy; MDG, mean decrease Gini; SVM, support vector machine; RDWCV, red cell distribution width-coefficient of variation; MCHC, mean corpuscular hemoglobin concentration; MPV, mean platelet volume; LPR, large platelet ratio; PDW, platelet distribution width; WBC, white blood cell count; HDL, high-density lipoprotein; VLDL, very low-density lipoprotein; LDL, low-density lipoprotein; GGT, gamma-glutamyl transferase; AST, aspartate aminotransferase; BMI, body mass index; MCH, mean corpuscular hemoglobin; ALT, alanine aminotransferase; ALP, alkaline phosphatase; USG, urine specific gravity; MCV, mean corpuscular volume; HGB, hemoglobin.

**Figure 1:**
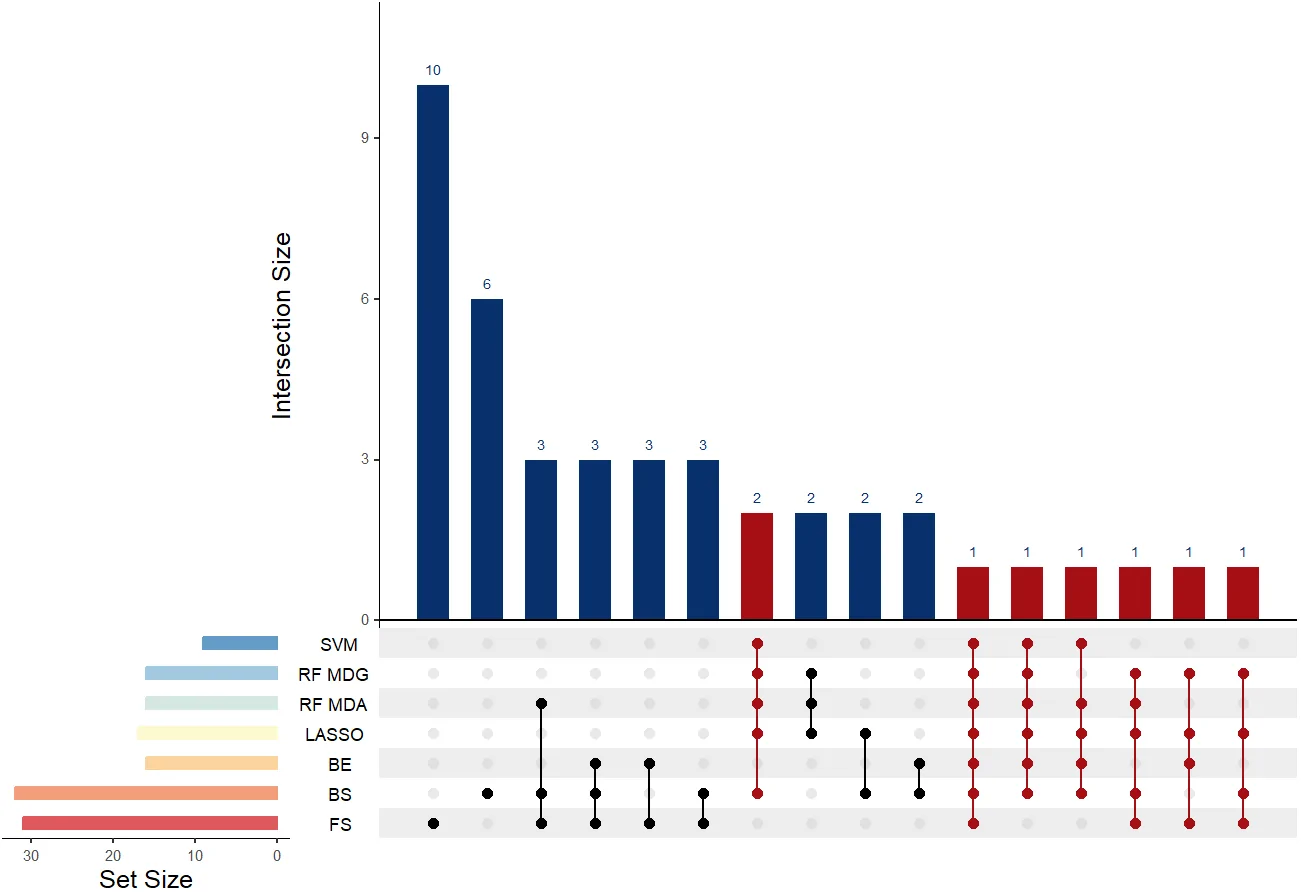
UpSet plot of the intersections of seven feature selection methods. Highlighted intersections (in red) correspond to the major overlaps of selected features across multiple methods. FS, stepwise regression-forward selection; BS, stepwise regression-backward selection; BE, stepwise regression-bidirectional elimination; LASSO, least absolute shrinkage and selection operator; RF-MDA, random forest-mean decrease accuracy; RF-MDG, random forest-mean decrease Gini; SVM, support vector machine.

### 3.3 Model performance

Model performance was assessed across three dimensions: discrimination, calibration, and clinical utility (**Figure 2**). The performance metrics of the osteoporosis prediction models are presented in **Table 3**. In the comparison of model discrimination performance, the AUC for the six prediction models ranged from 0.760 to 0.895. The RF and ANN models achieved higher AUC of 0.895 (95% CI, 0.867-0.923) and 0.890 (95% CI, 0.862-0.919), respectively, followed by the LR model with the AUC of 0.877 (95% CI, 0.847-0.907). The SVM model yielded the lowest AUC of 0.760 (95% CI, 0.721-0.799). In addition, the OSTA score yields an AUC of 0.864 (95% CI, 0.812-0.917). Pairwise DeLong’s tests revealed statistically significant differences in AUC values across all model pairs (P<0.001) except between RF and ANN (P = 0.052), suggesting comparable discriminative ability between these two models. At Youden thresholds, the models showed distinct trade-offs once translated into binary triage decisions. ANN achieved the highest sensitivity (0.969) and NPV (0.996), indicating strong rule-out performance. In contrast, XGB provided the highest accuracy (0.814) and PPV (0.245) with higher specificity (0.817), albeit with lower sensitivity (0.781) and NPV (0.980) compared with ANN. LR offered balanced performance profiles with competitive performance (AUC 0.877; ACC 0.768).

**Figure 2:**
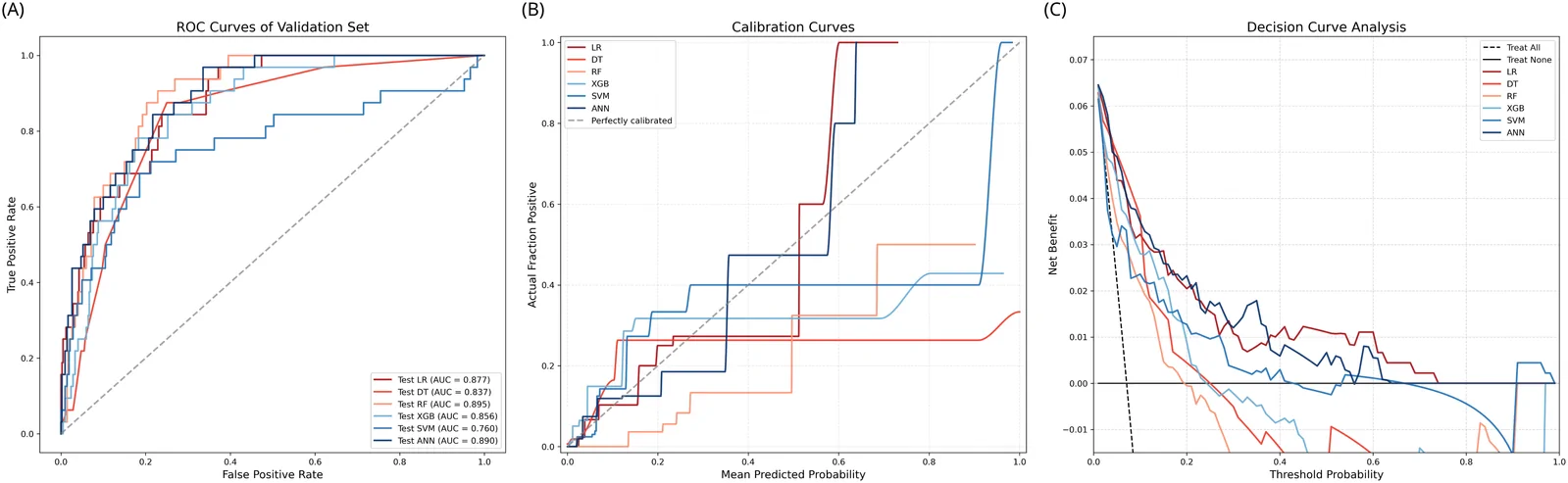
Performance evaluation of predictive models for osteoporosis in the validation set. **(A)** ROC curves, **(B)** calibration curves, and **(C)** decision curve analysis. LR, logistic regression; DT, decision tree; RF, random forest; XGB, extreme gradient boosting; SVM, support vector machine; ANN, artificial neuron network; ROC, receiver-operating curve; AUC, area under the curve.

**Table 3:** Performance metrics of all predictive models of validation set.

Model	LR	DT	RF	XGB	SVM	ANN
AUC (95% CI)	0.877 (0.847-0.907)	0.837 (0.803-0.871)	0.895 (0.867-0.923)	0.856 (0.823-0.888)	0.760 (0.721-0.799)	0.890 (0.862-0.919)
Cutoff	0.069	0.103	0.242	0.044	0.064	0.034
ACC (95% CI)	0.768 (0.727-0.804)	0.759 (0.717-0.796)	0.781 (0.741-0.817)	0.814 (0.776-0.847)	0.783 (0.743-0.819)	0.686 (0.642-0.727)
SEN (95% CI)	0.844 (0.681-0.930)	0.875 (0.718-0.949)	0.906 (0.757-0.966)	0.781 (0.611-0.889)	0.719 (0.545-0.844)	0.969 (0.842-0.993)
SPE (95% CI)	0.762 (0.719-0.800)	0.750 (0.706-0.789)	0.771 (0.729-0.809)	0.817 (0.777-0.851)	0.788 (0.746-0.824)	0.664 (0.618-0.708)
PLR (95% CI)	3.54 (2.82-4.45)	3.50 (2.83-4.32)	3.96 (3.22-4.88)	4.26 (3.24-5.60)	3.39 (2.55-4.51)	2.89 (2.49-3.35)
NLR (95% CI)	0.21 (0.09-0.46)	0.17 (0.07-0.42)	0.12 (0.04-0.36)	0.27 (0.14-0.52)	0.36 (0.20-0.62)	0.05 (0.01-0.32)
PPV (95% CI)	0.213 (0.151-0.292)	0.211 (0.150-0.288)	0.232 (0.167-0.314)	0.245 (0.172-0.337)	0.205 (0.141-0.290)	0.180 (0.130-0.245)
NPV (95% CI)	0.985 (0.965-0.993)	0.987 (0.968-0.995)	0.991 (0.974-0.997)	0.980 (0.959-0.990)	0.974 (0.950-0.986)	0.996 (0.980-0.999)
F1	0.340	0.339	0.369	0.373	0.319	0.304
Brier	0.051	0.070	0.078	0.066	0.059	0.051

LR, logistic regression; DT, decision tree; RF, random forest; XGB, extreme gradient boosting; SVM, support vector machine; ANN, artificial neuron network; AUC, area under receiver-operating curve; CI, confidence interval; ACC, accuracy; SEN, sensitivity; SPE, specificity; PLR, positive likelihood ratio; NLR, negative likelihood ratio; PPV, positive predictive value; NPV, negative predictive value.

Calibration analysis was performed to assess the agreement between predicted and observed probabilities across models. The calibration curves of the six models are shown in **Figure 2B**. The accuracy of probabilistic predictions was further quantified using Brier scores. The ANN and LR models achieved the lowest Brier scores of 0.051, indicating superior overall calibration. Notably, despite strong discrimination performance, the RF model showed the poorest calibration (Brier score = 0.078). Clinical utility and net benefit of each prediction model were estimated with DCA (**Figure 2C**). The ANN and LR models provided higher net benefit compared to other models, implying favorable clinical usefulness in decision-making.

Bootstrap performance distributions across models and feature sets were summarized in **Supplementary Table S2**. Across these conditions, the performance was stable across resample iterations.

### 3.4 Model interpretation

Benchmarking analysis combining multiple metrics demonstrated the ANN model’s optimal screening performance for osteoporosis prediction. To explain the ANN model, SHAP analysis was performed. SHAP values allow quantification of the contribution of each feature to individual predictions, thereby providing both global and local interpretability of complex models. As shown in **Figure 3A**, the SHAP bar plot of mean absolute values indicated that age and sex were the most influential predictor, followed by BMI, ALP, hematocrit, HDL, urea, and platelet count. The summary plot (**Figure 3B**) demonstrated the direction and distribution of feature effects across the dataset. Specifically, age, female sex, ALP, HDL, and urea are positively correlated with the risk of osteoporosis, while BMI, hematocrit, and platelet are negatively correlated.To further illustrate the predictions on individual level, force plot visualization was generated for two representative participants (**Figures 3C, D**). For example, in a participant classified as high risk (**Figure 3C**), the model output of risk prediction was driven by older age, female sex, lower BMI, elevated ALP, reduced hematocrit, higher HDL, elevated urea, and lower platelet count.

**Figure 3:**
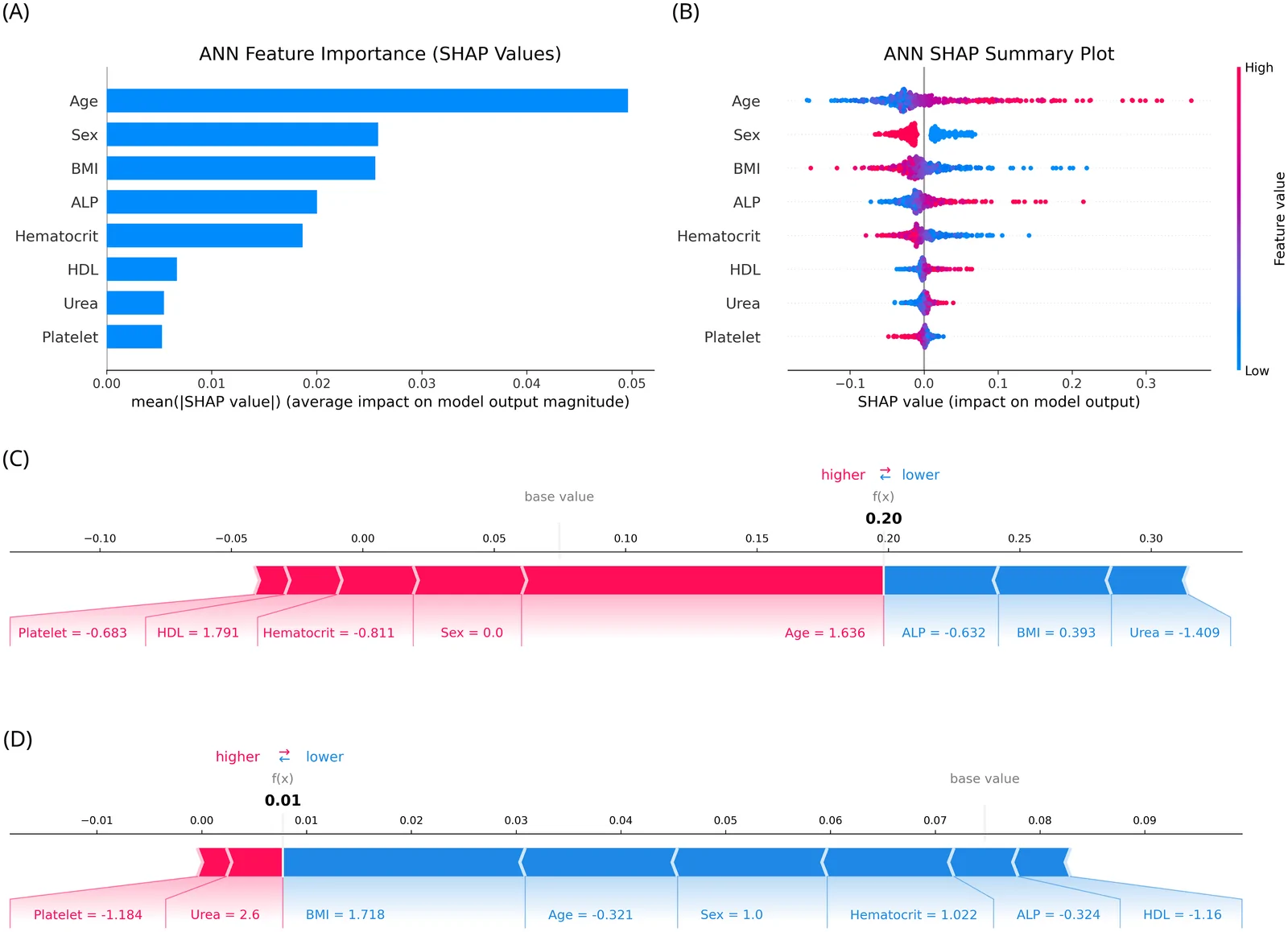
Interpretation of the ANN model for osteoporosis prediction. **(A)** Feature importance plot showing the ranking of mean absolute SHAP values of the key predictors. **(B)** SHAP summary plot illustrating the distribution and directionality of feature effects across participants. **(C)** Force plot of a representative high-risk participant. **(D)** Force plot of a representative low-risk participant. ANN, artificial neuron network; SHAP, SHapley Additive exPlanations; BMI, body mass index; ALP, alkaline phosphatase; HDL, high-density lipoprotein.

## 4 Discussion

The clinical significance of osteoporosis lies in the fractures that occur as a consequence of increased bone fragility. Osteoporosis-related fractures not only cause a sharp decline in patients’ quality of life, but also impose a significant social and economic burden (19). Despite consensus on the importance of early intervention to prevent osteoporotic fractures, reliable and cost-effective screening strategies that can be integrated into resource-limited healthcare settings to predict osteoporosis are critically lacking. This study developed a multiple ML algorithms-driven framework using basic health examination data, with the potential to facilitate large-scale osteoporosis screening.

ML holds significant promise to revolutionize osteoporosis management by enabling early detection, precise risk stratification, and tailored interventions. However, the current evidence is heterogeneous, often lacking sufficient benchmarking of model metrics and robust external validation (16). The selection of predictors for target outcomes is a crucial step in predictive model development. Unlike previous studies that relied on a limited set of feature selection strategies, this study incorporates seven ML methods to systematically identify predictors, ensuring the robustness and reproducibility of predictor choice. All predictors are derived from indicators in basic health examination, which are easily accessible and require no additional tests or expenses. By comparing six ML models on multi-dimensional metrics, the ANN model demonstrated superior screening performance that makes it a reliable and convenient tool in predicting osteoporosis. Notably, we provided transparent risk explanation via SHAP to enhance interpretability, bridging the gap between complex ML algorithms and clinical decision-making.

Eight variables that were selected by at least four of the seven feature selection methods were ultimately identified as relevant predictors, including age, sex, BMI, ALP, hematocrit, HDL, urea, and platelet count. In prior studies, advanced age and female sex had been recognized as risk factors for osteoporosis (20, 21), largely due to age-related bone loss and postmenopausal estrogen deficiency. Consistently, BMI were negatively correlated with the risk of osteoporosis (22). Increased mechanical loading on bones in individuals with higher body weight necessitates greater BMD for skeletal support. And reduced muscle mass or function might contribute to lower BMD by impairing balance and mobility (23, 24). ALP is essential in bone metabolism (25). Previous studies have established a positive association between elevated ALP levels and the risk of osteoporosis (22, 26), which is consistent with our findings, but the causal relationship remains uncertain. Hematocrit ranked among the top five predictors in the SHAP analysis, reflecting evidence that anemia contributes to disturbances in bone metabolism (27–29). Variables including HDL, urea, and platelet count emerged as significant predictors, aligned with findings from studies linking systemic metabolic and hematologic factors to bone remodeling and homeostasis (30–35).

Evaluation across multiple performance domains showed that the ANN model had superior ability to predict osteoporosis, with advantages in discrimination, calibration, and clinical net benefit. RF and ANN attained the highest AUCs (0.895 and 0.890), indicating superior risk stratification, yet RF exhibited the poorest Brier score among the models tested. This divergence underscores an important and clinically relevant trade-off between discrimination and calibration. ANN and LR yielded the lowest Brier scores and provided higher net benefit on decision-curve analysis across plausible threshold ranges, suggesting they may be more suitable when calibrated probabilities for threshold-based DXA triage are required. This framework is intended for primary-care triage prior to DXA, where the principal aim is to minimize missed osteoporosis cases while reducing unnecessary DXA referrals compared with a screen-all approach. In this context, the ANN performs well because its operating characteristics align with a screening workflow that prioritizes case capture. However, if a health system’s primary objective is to maximize the proportion of DXA-prioritized individuals who truly have osteoporosis (i.e., higher yield/PPV among those referred), then XGB may be preferred. LR remains a highly interpretable and well-calibrated option with competitive overall performance, and it may be the preferred choice when transparency and deployment feasibility are prioritized. Overall, this benchmarking highlights practical trade-offs between predictive performance, interpretability, and clinical workflow needs.

Traditional osteoporosis screening tools often rely on subjective variables and require additional patient-reported inputs, which may limit their applicability in real-world clinical workflows (8, 9). Tools such as FRAX and OSTA have shown modest discriminative ability in prior cohorts, with reported AUCs ranging from 0.536 to 0.796 and 0.700 to 0.874, respectively (36–38). In our study, OSTA yielded an AUC of 0.864 in the validation set. Notably, our ANN model achieved higher discrimination and outperformed the traditional tools. Moreover, unlike the increasing studies that applied ML to osteoporosis prediction, which relied on large numbers of heterogeneous predictors such as genetic factors and nutrient intake (11, 14, 21, 39, 40), our ANN model delivered comparable or better predictive performance while requiring fewer input variables. All predictors used are readily available from basic health examination panels without additional tests and cost, thereby reducing implementation barriers and facilitating integration of the algorithm into electronic health records. Notably, a recent study adopted a rigorous validation strategy by developing osteoporosis prediction models using routine clinical variables and evaluating the selected model not only in an internal test set (NHANES) but also in an independent external cohort, although the study focused exclusively on elderly women (15). The reported AUC of the selected model decreased from 0.805 in the internal test set to 0.740 in the external validation cohort, illustrating the potential impact of population and setting differences on model transportability.

Nonetheless, several limitations warrant consideration. First, this was a single-center study, and although internal validation was performed using a held-out test set, external validation in diverse populations and clinical settings is needed to confirm generalizability and transportability. To address this, future multicenter collaborations will be prioritized to validate and refine the model. Second, the cross-sectional nature of the dataset limits causal inference. In addition, progression of osteoporosis warrants longitudinal follow-up and analysis. Third, although we applied a consensus feature selection strategy to enhance robustness, the choice of included algorithms and cut-off criteria may influence the final predictor set. Future studies may explore automated feature selection pipelines or incorporate domain knowledge to refine feature inclusion.

We are exploring several avenues to enhance the real-world applicability of this framework in the future. Multicenter validation is essential to refine the model and enhance confidence in its use across heterogeneous healthcare settings. Integrating this framework into hospital electronic health record systems and routine health examination platforms could enable automated, real-time risk stratification to guide DXA referral and treatment decisions. Embedding the model within existing clinical workflows would minimize additional clinician workload and facilitate point-of-care decision support. In addition, incorporating adaptive learning capabilities would allow the model to evolve over time. Each new prediction could feed back into the database, serving both as an input for subsequent predictions and as a calibration point for iterative model refinement. This continuous learning loop would progressively improve predictive performance.

## 5 Conclusion

In this study, we present a robust, interpretable, and low-cost ML framework for osteoporosis prediction that leverages routine health examination data and consensus feature selection strategy. Rigorous model evaluation demonstrated that ANN model provided a wider net. The proposed framework may support pre-DXA triage, subject to independent external validation, and potentially facilitate earlier detection of osteoporosis in primary care and resource-constrained settings.

## Data Availability

The raw data supporting the conclusions of this article will be made available by the authors, without undue reservation.
