# Supplementary material for: A primary care-oriented framework for osteoporosis prediction: integrating multi-algorithm feature selection and machine learning model benchmarking
Source: Front Endocrinol (Lausanne). 2026 Oct 5;17:1917386. doi: 10.3389/fendo.2026.1917386 (PMC13677370; doi:10.3389/fendo.2026.1917386)
Supplement: Supplementary file 1 [file Table1.docx]

**Supplementary material**

Table S1. Descriptive statistics of training and validation sets

|  | Training set  (n=1808) | Testing set  (n=452) | p |
| --- | --- | --- | --- |
| Osteoporosis, n (%) | 128 (7.1) | 32 (7.1) | 1.000 |
| Age (years) | 55.00 (48.00, 61.00) | 55.00 (48.00, 61.00) | 1.000 |
| Sex, n (%) |  |  |  |
| Female | 847 (46.8) | 200 (44.2) | 0.343 |
| Male | 961 (53.2) | 252 (55.8) |  |
| BMI (kg/m^2) | 23.87 (21.88, 26.01) | 24.21 (22.17, 26.26) | 0.126 |
| Hypertension, n (%) | 443 (24.5) | 107 (23.7) | 0.759 |
| RBC (x10^12/L) | 4.78 (4.44, 5.10) | 4.72 (4.43, 5.09) | 0.384 |
| HGB (g/L) | 142.00 (132.00, 152.00) | 141.50 (131.00, 151.00) | 0.694 |
| MCV (fL) | 90.50 (87.60, 93.10) | 90.70 (88.10, 92.80) | 0.491 |
| MCH (pg) | 30.08 (29.00, 31.00) | 30.10 (29.20, 31.00) | 0.238 |
| MCHC (g/L) | 330.60 (323.00, 338.00) | 332.00 (325.00, 339.00) | 0.034 |
| Hematocrit (%) | 0.43 (0.40, 0.45) | 0.43 (0.40, 0.45) | 0.723 |
| Platelet (×10^9/L) | 241.00 (208.00, 279.00) | 245.00 (207.00, 282.00) | 0.526 |
| Plateletcrit (%) | 0.24 (0.21, 0.27) | 0.24 (0.21, 0.27) | 0.671 |
| MPV (fL) | 9.80 (9.34, 10.40) | 9.70 (9.30, 10.20) | 0.020 |
| WBC (×10^9/L) | 5.89 (5.03, 6.91) | 5.90 (5.01, 6.97) | 0.706 |
| Lymphocyte (×10^9/L) | 1.99 (1.63, 2.38) | 1.99 (1.61, 2.37) | 0.709 |
| Neutrophil (×10^9/L) | 3.20 (2.59, 4.00) | 3.29 (2.67, 4.02) | 0.431 |
| Eosinophil (×10^9/L) | 0.14 (0.09, 0.22) | 0.14 (0.08, 0.22) | 0.846 |
| Basophil (×10^9/L) | 0.03 (0.02, 0.04) | 0.03 (0.02, 0.04) | 0.860 |
| Monocyte (×10^9/L) | 0.41 (0.33, 0.51) | 0.41 (0.33, 0.52) | 0.911 |
| Na (mmol/L) | 139.40 (138.60, 140.00) | 139.40 (138.80, 140.20) | 0.729 |
| K (mmol/L) | 4.10 (3.95, 4.23) | 4.10 (3.93, 4.26) | 0.934 |
| Ca (mmol/L) | 2.36 (2.31, 2.41) | 2.36 (2.31, 2.40) | 0.811 |
| Cl (mmol/L) | 104.06 (103.00, 105.20) | 104.20 (103.00, 105.20) | 0.374 |
| Uric Acid (μmol/L) | 368.00 (308.00, 432.00) | 371.00 (314.30, 437.50) | 0.270 |
| Creatinine (μmol/L) | 73.00 (61.00, 85.00) | 73.50 (62.00, 86.00) | 0.294 |
| Urea (mmol/L) | 5.09 (4.32, 5.92) | 5.15 (4.39, 5.94) | 0.694 |
| Glucose (mmol/L) | 5.14 (4.75, 5.67) | 5.12 (4.74, 5.71) | 0.981 |
| Total Cholesterol (mmol/L) | 5.32 (4.66, 6.04) | 5.36 (4.66, 5.96) | 0.832 |
| Triglycerides (mmol/L) | 1.31 (0.96, 1.86) | 1.31 (0.99, 1.85) | 0.815 |
| HDL (mmol/L) | 1.35 (1.16, 1.58) | 1.34 (1.12, 1.55) | 0.292 |
| LDL (mmol/L) | 3.40 (2.90, 3.89) | 3.44 (2.90, 3.91) | 0.959 |
| Total Bilirubin (μmol/L) | 13.19 (10.70, 16.10) | 13.20 (11.30, 16.33) | 0.223 |
| Direct Bilirubin (μmol/L) | 2.09 (1.60, 2.60) | 2.08 (1.70, 2.60) | 0.347 |
| Indirect Bilirubin (μmol/L) | 11.00 (8.90, 13.60) | 11.20 (9.50, 13.62) | 0.202 |
| GGT (U/L) | 20.00 (14.00, 30.00) | 20.00 (14.00, 34.00) | 0.223 |
| AST (U/L) | 21.00 (18.00, 26.00) | 21.00 (18.00, 25.00) | 0.970 |
| ALT (U/L) | 19.00 (15.00, 27.00) | 19.00 (15.00, 27.00) | 0.792 |
| ALP (U/L) | 71.30 (61.00, 84.10) | 71.00 (60.00, 86.00) | 0.721 |
| Total Protein (g/L), mean (SD) | 72.80 (3.67) | 72.83 (3.65) | 0.874 |
| Albumin (g/L) | 44.50 (42.42, 46.60) | 44.40 (42.40, 46.20) | 0.352 |
| Globulin (g/L) | 28.20 (25.80, 30.70) | 28.33 (26.00, 31.10) | 0.465 |
| Urine PH | 5.50 (5.00, 6.50) | 5.50 (5.50, 6.50) | 0.485 |
| Urine Protein (positive), n (%) | 28 (1.5) | 7 (1.5) | 1.000 |
| Urine Bilirubin (positive), n (%) | 12 (0.7) | 1 (0.2) | 0.485 |
| Urobilinogen (positive), n (%) | 53 (2.9) | 21 (4.6) | 0.076 |
| Urine Glucose (positive), n (%) | 86 (4.8) | 25 (5.5) | 0.468 |
| Urine Nitrite (positive), n (%) | 30 (1.7) | 9 (2.0) | 0.686 |
| Urine Occult Blood (positive), n (%) | 474 (26.2) | 129 (28.5) | 0.341 |
| Urine Ketone Bodies (positive), n (%) | 34 (1.9) | 4 (0.9) | 0.157 |
| Urine WBC (positive), n (%) | 429 (23.7) | 108 (23.9) | 0.951 |

Data are expressed as median (IQR) unless otherwise indicated.

Abbreviations: BMI, body mass index; RBC, red blood cell; HGB, hemoglobin; MCV, mean corpuscular volume; MCH, mean corpuscular hemoglobin; MCHC, mean corpuscular hemoglobin concentration; MPV, mean platelet volume; WBC, white blood cell; HDL, high-density lipoprotein; VLDL, very-low-density lipoprotein; LDL, low-density lipoprotein; GGT, gamma-glutamyl transferase; AST, aspartate aminotransferase; ALT, alanine aminotransferase; ALP, alkaline phosphatase

Table S2. Bootstrap performance distributions across models and feature sets

| Feature set | Model | AUC (95% CI) | ACC (95% CI) | SEN (95% CI) | SPE (95% CI) | PPV (95% CI) | NPV (95% CI) | F1 (95% CI) | Brier (95% CI) |
| --- | --- | --- | --- | --- | --- | --- | --- | --- | --- |
| ≥3/7 | LR | 0.877 (0.823-0.917) | 0.817 (0.653-0.892) | 0.829 (0.687-0.969) | 0.816 (0.631-0.905) | 0.271 (0.162-0.368) | 0.985 (0.973-0.997) | 0.403 (0.276-0.505) | 0.052 (0.046-0.061) |
| ≥3/7 | DT | 0.802 (0.722-0.879) | 0.800 (0.652-0.863) | 0.750 (0.594-0.906) | 0.804 (0.628-0.879) | 0.235 (0.151-0.308) | 0.977 (0.965-0.991) | 0.355 (0.257-0.439) | 0.064 (0.055-0.074) |
| ≥3/7 | RF | 0.882 (0.834-0.924) | 0.790 (0.721-0.859) | 0.878 (0.719-0.970) | 0.782 (0.704-0.862) | 0.245 (0.188-0.319) | 0.991 (0.981-1.000) | 0.383 (0.311-0.472) | 0.072 (0.064-0.082) |
| ≥3/7 | XGB | 0.843 (0.784-0.891) | 0.752 (0.641-0.847) | 0.844 (0.688-0.969) | 0.745 (0.618-0.853) | 0.208 (0.153-0.281) | 0.985 (0.971-0.996) | 0.331 (0.258-0.414) | 0.059 (0.050-0.069) |
| ≥3/7 | SVM | 0.799 (0.723-0.855) | 0.797 (0.663-0.907) | 0.732 (0.563-0.876) | 0.802 (0.643-0.926) | 0.236 (0.155-0.396) | 0.976 (0.963-0.987) | 0.350 (0.259-0.487) | 0.058 (0.052-0.066) |
| ≥3/7 | ANN | 0.876 (0.822-0.919) | 0.778 (0.670-0.887) | 0.904 (0.781-1.000) | 0.771 (0.648-0.895) | 0.236 (0.166-0.362) | 0.988 (0.975-0.997) | 0.368 (0.282-0.491) | 0.054 (0.047-0.063) |
| ≥4/7 | LR | 0.872 (0.812-0.926) | 0.798 (0.648-0.903) | 0.833 (0.688-0.969) | 0.804 (0.643-0.912) | 0.257 (0.160-0.403) | 0.985 (0.974-0.997) | 0.385 (0.274-0.532) | 0.053 (0.046-0.060) |
| ≥4/7 | DT | 0.818 (0.716-0.895) | 0.798 (0.648-0.872) | 0.776 (0.625-0.938) | 0.800 (0.628-0.881) | 0.237 (0.157-0.315) | 0.979 (0.966-0.993) | 0.360 (0.267-0.451) | 0.061 (0.051-0.071) |
| ≥4/7 | RF | 0.877 (0.820-0.926) | 0.778 (0.695-0.876) | 0.836 (0.688-0.969) | 0.769 (0.674-0.883) | 0.237 (0.178-0.333) | 0.990 (0.979-1.000) | 0.372 (0.296-0.488) | 0.080 (0.070-0.091) |
| ≥4/7 | XGB | 0.844 (0.782-0.898) | 0.778 (0.644-0.885) | 0.821 (0.656-0.938) | 0.774 (0.621-0.902) | 0.227 (0.149-0.333) | 0.983 (0.971-0.994) | 0.352 (0.257-0.456) | 0.059 (0.050-0.069) |
| ≥4/7 | SVM | 0.738 (0.633-0.828) | 0.839 (0.692-0.923) | 0.613 (0.375-0.813) | 0.856 (0.695-0.960) | 0.269 (0.155-0.452) | 0.967 (0.953-0.981) | 0.363 (0.249-0.500) | 0.058 (0.052-0.067) |
| ≥4/7 | ANN | 0.880 (0.820-0.929) | 0.807 (0.666-0.903) | 0.900 (0.780-1.000) | 0.795 (0.626-0.914) | 0.268 (0.170-0.400) | 0.985 (0.974-0.997) | 0.398 (0.284-0.542) | 0.051 (0.045-0.059) |
| ≥5/7 | LR | 0.872 (0.810-0.922) | 0.784 (0.659-0.887) | 0.850 (0.688-0.969) | 0.779 (0.638-0.895) | 0.243 (0.164-0.362) | 0.986 (0.973-0.997) | 0.372 (0.277-0.495) | 0.053 (0.046-0.060) |
| ≥5/7 | DT | 0.822 (0.739-0.895) | 0.800 (0.663-0.865) | 0.779 (0.625-0.938) | 0.802 (0.645-0.879) | 0.240 (0.159-0.313) | 0.980 (0.968-0.993) | 0.364 (0.267-0.452) | 0.061 (0.051-0.070) |
| ≥5/7 | RF | 0.872 (0.814-0.922) | 0.782 (0.699-0.867) | 0.852 (0.688-0.969) | 0.773 (0.679-0.869) | 0.237 (0.182-0.333) | 0.990 (0.980-1.000) | 0.373 (0.304-0.479) | 0.086 (0.075-0.099) |
| ≥5/7 | XGB | 0.854 (0.790-0.902) | 0.763 (0.635-0.861) | 0.859 (0.718-0.969) | 0.756 (0.612-0.867) | 0.219 (0.155-0.312) | 0.986 (0.973-0.997) | 0.346 (0.259-0.449) | 0.060 (0.050-0.069) |
| ≥5/7 | SVM | 0.723 (0.620-0.808) | 0.815 (0.639-0.909) | 0.606 (0.406-0.781) | 0.831 (0.628-0.943) | 0.234 (0.138-0.385) | 0.965 (0.953-0.978) | 0.328 (0.230-0.447) | 0.060 (0.054-0.067) |
| ≥5/7 | ANN | 0.878 (0.819-0.924) | 0.797 (0.690-0.894) | 0.895 (0.781-1.000) | 0.793 (0.671-0.907) | 0.256 (0.178-0.375) | 0.986 (0.974-0.997) | 0.387 (0.296-0.509) | 0.052 (0.046-0.060) |

Footnote: ≥3/7, ≥4/7 and ≥5/7 denote the predictor sets comprising the variables retained by at least three, at least four, or at least five of the seven feature-selection methods, corresponding to 24, 8 and 6 variables, respectively.

Abbreviations: LR, logistic regression; DT, decision tree; RF, random forest; XGB, extreme gradient boosting; SVM, support vector machine; ANN, artificial neuron network; AUC, area under receiver-operating curve; CI, confidence interval; ACC, accuracy; SEN, sensitivity; SPE, specificity; PPV, positive predictive value; NPV, negative predictive value
